# Corticosterone and glucose are correlated and show similar response patterns to temperature and stress in a free-living bird

**DOI:** 10.1242/jeb.246905

**Published:** 2024-07-24

**Authors:** Paola M. Millanes, Lorenzo Pérez-Rodríguez, Juan G. Rubalcaba, Diego Gil, Blanca Jimeno

**Affiliations:** ^1^ Departamento de Biología y Geología, Física y Química Inorgánica, Universidad Rey Juan Carlos, 28933 Móstoles, Madrid, Spain; ^2^ Instituto de Investigación en Recursos Cinegéticos (IREC), CSIC-UCLM-JCCM, Ronda de Toledo 12, 13005 Ciudad Real, Spain; ^3^ Department of Biodiversity, Ecology and Evolution, Faculty of Biological Sciences. Complutense University of Madrid, José Antonio Novais, 12, 28040 Madrid, Spain; ^4^ Department of Evolutionary Ecology, Museo Nacional de Ciencias Naturales (MNCN-CSIC), José Gutiérrez Abascal, 2, 28006 Madrid, Spain; ^5^ Pyrenean Institute of Ecology (IPE-CSIC), Avda Nuestra Señora de la Victoria, s/n, 22700 Jaca, Huesca, Spain

**Keywords:** Glucocorticoids, Energy expenditure, Stress response, Metabolic rate, *Sturnus unicolor*

## Abstract

Glucocorticoid (GC) hormones have traditionally been interpreted as indicators of stress, but the extent to which they provide information on physiological state remains debated. GCs are metabolic hormones that amongst other functions ensure increasing fuel (i.e. glucose) supply on the face of fluctuating energetic demands, a role often overlooked by ecological studies investigating the consequences of GC variation. Furthermore, because energy budget is limited, in natural contexts where multiple stimuli coexist, the organisms' ability to respond physiologically may be constrained when multiple triggers of metabolic responses overlap in time. Using free-living spotless starling (*Sturnus unicolor*) chicks, we experimentally tested whether two stimuli of different nature known to trigger a metabolic or GC response, respectively, cause a comparable increase in plasma GCs and glucose. We further tested whether response patterns differed when both stimuli occurred consecutively. We found that both experimental treatments caused increases in GCs and glucose of similar magnitude, suggesting that both variables fluctuate along with variation in energy expenditure, independently of the trigger. Exposure to the two stimuli occurring subsequently did not cause a difference in GC or glucose responses compared with exposure to a single stimulus, suggesting a limited capacity to respond to an additional stimulus during an ongoing acute response. Lastly, we found a positive and significant correlation between plasma GCs and glucose after the experimental treatments. Our results add to the increasing research on the role of energy expenditure on GC variation, by providing experimental evidence on the association between plasma GCs and energy metabolism.

## Teaser

**Summary:** Experimental evidence shows that two stimuli triggering a metabolic (temperature decrease) or glucocorticoid (restraint) response caused comparable increases in plasma glucocorticoids and glucose.

## INTRODUCTION

In an everchanging world, understanding the effects of environmental fluctuations on wild populations is fundamental to predict the consequences of anthropogenic perturbations and the capacity of the organisms to cope (Dantzer et al., 2014; Madliger et al., 2015). On a daily basis, animals respond to predictable and unpredictable environmental changes through a set of physiological and behavioral processes (Bobba-Alves et al., 2022). One of the key mechanisms mediating environmental coping and homeostasis maintenance in vertebrates is the activation of the hypothalamic–pituitary–adrenal (HPA) axis, which results in the release of glucocorticoid hormones into the bloodstream (Dantzer et al., 2014; Jimeno et al., 2018; Tsigos and Chrousos, 2002; Burford et al., 2017; Nicolaides et al., 2015). Glucocorticoids (GCs; e.g. corticosterone, cortisol) are thus widely considered to be mediators of physiological responses in the face of environmental change (Sapolsky et al., 2000; McEwen and Wingfield, 2003; Koolhaas et al., 2011; Madliger and Love, 2016).

GC concentrations in plasma oscillate daily and seasonally along with energy demands and activity cycles (i.e. baseline concentrations; Marasco et al., 2015; Madliger et al., 2015; Landys et al., 2006). GCs also increase rapidly, in the form of an acute response (i.e. ‘stress response’), when the organism is exposed to unexpected stimuli that pose a threat to homeostasis (i.e. ‘stressors’; McEwen and Wingfield, 2003; Romero and Wingfield, 2015; Romero and Butler, 2007; Sapolsky et al., 2000), to then return to baseline levels through negative feedback mechanisms (Malisch et al., 2010). These response patterns to external stimuli have contributed to GCs being widely known as ‘stress hormones’ and used as a proxy of individual condition or animal welfare (Madliger et al., 2015; Madliger and Love, 2014; McCormick and Romero, 2017). However, this interpretation, as well as the use and definition of the term ‘stress’, remains questioned in the field of ecology (Koolhaas et al., 2011; McDougall-Shackleton et al., 2019; Jimeno and Verhulst, 2023). Furthermore, the generality of an association between plasma GCs and fitness components is poorly supported by the literature (Marasco et al., 2015; Nelson et al., 2015; Madliger and Love, 2016; Petrullo et al., 2022; Patterson et al., 2014), and ecological and evolutionary physiologists have long advocated the need to reconsider the causes of GC variation from a wider, more mechanistic perspective (Koolhaas et al., 2011; Landys et al., 2006; McEwen and Wingfield, 2003; McDougall-Shackleton et al., 2019). GCs are primarily metabolic hormones and, amongst other actions, mediate glucose synthesis and mobilization – by promoting gluconeogenesis in the liver and acting antagonistically to insulin in peripheral tissues decreasing glucose uptake – making it available for tissues and organs to cope with prevailing energetic needs (Romero and Beattie, 2021; Romero and Butler, 2007; Taff et al., 2022; Jimeno and Verhulst, 2023). GCs are thus expected to fluctuate with metabolic demands, facilitating resource allocation and availability on the face of perceived (‘reactive’ response) or anticipated (‘anticipatory’ response) increases in energy expenditure (Koolhaas et al., 2011; Herman, 2022), or restoring glucose levels following an adrenaline-supported burst of energy expenditure (Koolhaas et al., 2011). This metabolic source of GC variation, although widely assumed, is barely accounted for when quantifying and interpreting GC levels in an ecological context.

Environmental fluctuations are expected to be associated with a change in energetic demands to meet and overcome the challenge (Madliger et al., 2015; Jimeno and Verhulst, 2023). GC responses may thus be interpreted as a change in the rate at which the organism operates (Landys et al., 2006; Jimeno and Verhulst, 2023). Indeed, increasing evidence supports the role of energy expenditure as fundamental driver of GC variation (Koolhaas et al., 2011; McDougall-Shackleton et al., 2019; Landys et al., 2006), including some studies empirically showing an association between GCs and metabolic rate (Beerling et al., 2011; Buwalda et al., 2012; Malkoc et al., 2021; Jimeno et al., 2018; Rubalcaba and Jimeno, 2022; Jimeno and Verhulst, 2023). According to this framework, an increase in energetic needs would lead to increased plasma GCs and glucose mobilization (e.g. by reducing blood glucose uptake by tissues; Romero and Beattie, 2021; Jimeno and Verhulst, 2023) and synthesis (i.e. by inhibition of glycogen synthesis in the liver and facilitation of gluconeogenesis; Romero and Butler, 2007, Jimeno and Verhulst, 2023), along with a variety of downstream effects (Sapolsky et al., 2000; Landys et al., 2006; Jimeno and Verhulst, 2023). Despite this prediction – and in contrast to GC responses – a wide variation and context dependence of glucose responses to standardized stress have been reported. This literature shows that the magnitude of the response, and whether it occurs at all, is influenced by life history stage, season, time of day or diet (Remage-Healey and Romero, 2000; Deviche et al., 2016a,b). This may be explained by the complexity of glucose regulation, which involves multiple overlapping processes acting both in the short and longer terms. During an acute response, catecholamines act quickly and increase within seconds to induce energy (Herman, 2022; Romero and Beattie, 2021; Sapolsky et al., 2000). The GC response lags in time and lasts substantially longer (Herman, 2022), enhancing and prolonging the increase in blood glucose (Nonogaki, 2000; Romero and Beattie, 2021), or recovering energy stores after a brief burst of activity (Koolhaas et al., 2011). Interestingly, the association between GC and glucose levels has rarely been tested in an ecological context, and few studies have investigated whether between-individual variation in GC absolute levels or magnitude of change during an acute stress response predicts the degree of change in glucose availability (Deviche et al., 2016a,b; Jimeno et al., 2018; Taff et al., 2022). To accurately address the relationship between GCs and glucose in a context of variation in energy expenditure, it is essential to study GC and glucose variation simultaneously (Taff et al., 2022; Deviche et al., 2016a,b), in response to a variety of stimuli, and in different physiological states.

There is a wide variety of environmental factors triggering variation in energy expenditure. In endotherms, thermoregulation is a physiological process requiring a high, sustained energy investment, thus also driving GC variation (Jessop et al., 2016; Jimeno et al., 2018; Rubalcaba and Jimeno, 2022; Malkoc et al., 2021). In addition, within the daily environmental variation experienced by organisms, several unpredicted stimuli triggering an acute physiological response may overlap in time or occur subsequently. In this case, the ability of organisms to (metabolically) mount an adjusted response to several co-occurring disturbances may be constrained. For instance, a limited energy budget can constrain the ability of organisms to respond to multiple, overlapping energy-demanding activities over time. In these cases, organisms are expected to prioritize energy allocation into acute responses that help them overcome sudden challenges, limiting their investment in certain physiological processes. This limitation could be reflected in the level of GCs if such response facilitates metabolic rate (Jimeno and Verhulst, 2023). Improving our understanding of potential interactions among environmental factors triggering an acute physiological response is critical towards predicting organismal responses to increasingly fluctuating environments, because those stimuli will often co-occur in time and space (Simmons et al., 2021).

This work investigates whether two stimuli of different nature known to increase metabolic rate and plasma GCs, respectively (i.e. an experimental decrease in ambient temperature and a standardized ‘handling and restraint’ protocol), trigger a comparable response in plasma corticosterone (the main bird GC) and glucose in free-living spotless starling (*Sturnus unicolor*) chicks (14–16 days old). In the experiment we also included two control treatments to account for the effects that visiting the nest box and manipulating individuals may have on the two study variables. We predicted that both stimuli, independently of their nature, would trigger a GC response to meet increased energetic demands, reflected in increased plasma glucose. Because our interpretation of the predicted metabolic–GC association is based on the assumption that GCs ensure increasing fuel (i.e. glucose) supply to match higher energetic needs, we also tested correlations between glucose and corticosterone levels within and among individuals.

We additionally tested whether the corticosterone and glucose response patterns changed when both stimuli occur subsequently, in order to detect whether the ability of organisms to (metabolically) mount an adjusted response to several co-occurring disturbances is constrained. We predicted that an adjusted physiological response to the second stimulus may be constrained (i.e. being either absent or attenuated) by the ongoing acute response triggered by the first stimulus.

## MATERIALS AND METHODS

### Study system

This experiment was carried out in a long-term monitored (i.e. since 2004) spotless starling (*Sturnus unicolor* Temminck 1820) population located in central Spain (Soto del Real, Madrid; 40.7503°N, −3.8028°W). The study area is characterized by an open woodland of oak (*Quercus pyrenaica*) and ash (*Fraxinus angustifolia*) and the presence of grazing livestock. In this area, 246 nest boxes are monitored each breeding season (March–July). The present study was carried out during the first clutches of the 2022 breeding season.

The spotless starling is a non-migratory passerine that is distributed along the western Mediterranean. It is a semi-colonial, secondary cavity nester that readily occupies nest boxes, and defends a small area around the nest site against conspecifics. The species reproduces between April and July, laying two clutches during the reproductive season. Clutches contain between 3 and 6 eggs per nest (mean±s.d.: 4.72±0.57; Muriel et al., 2015). The chicks remain in the nest for approximately 20 days. Both sexes reach sexual maturity as 1-year-olds. However, in contrast to females, males rarely breed as 1-year-olds, most recruiting into the breeding population as 2- or 3-year-old adults (Veiga, 2002; Redondo et al., 2022).

All experimental procedures were carried out under the approval of the Animal Experimentation Ethical Committee of the University of Castilla La Mancha and the Council of Agriculture, Environment and Rural Development of Castilla-La Mancha (procedure number 010474/8-2022). Methods were carried out in accordance with these approved guidelines and regulations.

### Experimental design and sampling

During the 2022 breeding period, as part of the annual field monitoring, nest boxes were checked periodically from the start of the reproductive season to chick independence, which provided information on laying date, clutch size, hatching date and brood size. The study was carried out on chicks between 12 and 16 days of age (between 14 and 16 days for experimental treatments, see below). The design included four treatment groups (two control, two experimental), with a total sample size of 65 chicks in 17 nests. Because each chick was assigned to a single treatment, only clutches that had four chicks or more were included in the study, so that the four experimental groups were represented in each nest. This was true for all broods but three, where one of the nestlings of each brood died during the study, but before experimental manipulations, owing to natural causes (brood reduction is common in this species).

**Fig. 1.**
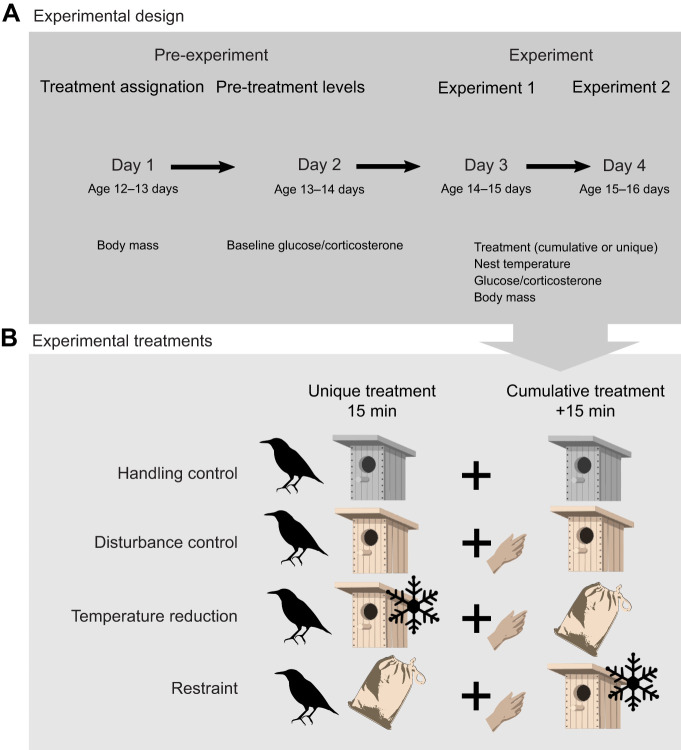
**Schematic representation of the experimental design.** (A) Experimental design and measurements taken each day and (B) the experimental treatments carried out on days 3 and 4. Experimental manipulations entailed a ‘unique treatment’ day in which individuals were subjected to their corresponding treatment for 15 min, and another day of ‘cumulative treatment’, in which individuals were subjected to the treatment for 30 min (15+15 min; see Materials and Methods). The experimental treatments performed were: (1) handling control: the chick remained undisturbed; (2) disturbance control: the chick was transferred to a nest box similar to the one used in the temperature reduction treatment to mimic experimental conditions; (3) temperature reduction treatment; and (4) restraint treatment. Uncolored nest boxes represent the original nest box, whereas colored ones represent the experimental and disturbance control ones (which were identical except for the presence of a temperature manipulation device in the experimental ones). The hand symbol represents the handling performed on the chicks after the first 15 min. For details, see Materials and Methods.

The experiment lasted 4 days, of which the first two (pre-treatment) corresponded to individual selection and marking and collection of baseline blood samples, respectively, and the last two (post-treatment) corresponded to the experimental treatments. On day 1 (age 12 or 13 days), individuals were weighed and marked with numbered metal rings. Additionally, they were individually marked on the head with waterproof ink markers, allowing a quick identification for baseline sample collection and experimental treatment allocation on days 3 and 4 (see below; Fig. 1). On day 2 (age 13 or 14 days), 150 μl of blood was drawn from the jugular vein in less than 3 min after first nest disturbance (i.e. lowering the nest box; Romero and Reed, 2005), to determine baseline glucose and corticosterone levels, in order to obtain individual reference values prior to experimental manipulation (i.e. pre-treatment) (Fig. 1). Time from first disturbance to the blood sample collection for each individual was recorded. Glucose levels were quantified with a blood glucose meter (Sinocare Safe AQ; test range=1.1–33.3 mmol l^−1^) from a drop of the freshly collected sample. Birds maintain nearly two-fold higher glucose levels than mammals of similar body mass apparently without experiencing detrimental effects, and are resistant to insulin-mediated glucose uptake into tissues, showing adaptations that enable them to thrive in a state of relative hyperglycemia (Braun and Sweazea, 2008; Sweazea, 2022). The repeatability of glucose measurements (within blood sample) was 97.7%. Experimental manipulations were carried out on days 3 (age 14 or 15 days) and 4 (age 15 or 16 days) (Fig. 1). These consisted of a ‘unique treatment’ day in which individuals were subjected to their corresponding treatment for 15 min, and a ‘cumulative treatment’ day, in which individuals were subjected to treatment for 30 min (15+15 min). During these 30 min, individuals in the two control groups remained in the same treatment, whereas those in the two experimental groups underwent 15 min of the experimental treatment experienced the previous day, and then another 15 min of the other experimental treatment (see details below). For each nest, nestlings were assigned to each treatment group at random across their brood size hierarchy. Also, the order in which the unique or cumulative treatments were performed was randomized, so that the same order was followed by all chicks within the nest, but the same number of nests underwent the unique treatment on day 3 and the cumulative treatment on day 4 or vice versa. Initial temperature inside the nest box (i.e. before experimental manipulations) was measured with a non-contact infrared thermometer (IDOIT HW - F7) on days 2, 3 and 4, in less than 1 min after first disturbance. In order to make these measurements consistent, we targeted each of the three inner sides of the nest box – excluding the side with the entrance – placing the thermometer at ∼10 cm distance, and at about half of the nest box height. We avoided taking measurements of the bottom of the nest box, which would be highly influenced by the number of chicks and their body size and temperature. We averaged these three measurements, and that was the temperature used in our analyses. This provided a measurement of the ambient temperature to which the chicks were being exposed, and which may influence baseline glucose and corticosterone levels (Jessop et al., 2016; Montoya et al., 2018; Jimeno et al., 2018). We also measured chick body surface temperature on the bare skin of the belly, on days 3 and 4 (experimental treatments) before and after the manipulations, to quantify the effect of the temperature reduction device (see below).

The four experimental manipulations carried out during days 3 and 4 were the following: (1) handling control, (2) disturbance control, (3) temperature reduction and (4) handling and restraint.

In the handling control, the chick remained in the original nest box, undisturbed, for 15 min during the unique treatment and 30 min during the cumulative treatment.

In the disturbance control, the chick was transferred to a nest box identical to the one used in the temperature reduction treatment (see below), but without additional manipulation. On the day of cumulative treatment, after 15 min, the chick was picked up/handled and returned to the same box, to match the handling disturbance of the experimental treatments (see below). This treatment thus showed the effect of additional disturbance (i.e. experimental treatments) as compared with handling effects only (i.e. handling control). Thus, it is the comparison between handling control and disturbance control that informed us about the increases in corticosterone and glucose that are caused by chick handling per se.

In the temperature reduction treatment, the chick was taken out of the nest box and placed in a similar nest box equipped with a temperature manipulation device. This device consisted of a Peltier plate, a thermometer, a power regulator and a ventilator to dissipate the heat generated by the plate. We used this device to gradually reduce the temperature inside the box down to 7°C (7.03±0.69°C mean±s.d.), which was within the range of the minimum temperatures recorded in our population during this period of the year and at the time of the day when the experiment was performed (P.M.M. and B.J., personal observation). Along with this decrease in ambient temperature, we registered a reduction of the chick’s surface temperature of −6.91±0.94°C (mean±s.d.) that was significantly stronger than that experienced by nestlings of the other treatments (β=−6.35, *F*=19.23, *P*<0.0001, d.f.=24.49; Fig. S1a,b). Because birds at this stage of development are mostly or fully endotherms (Price and Dzialowski, 2018), this decrease in surface temperature would increase the temperature gradient between the body core and surface, thus increasing heat dissipation and therefore metabolic investment. During the cumulative treatment, the chicks spent 15 min in the cold treatment, and were subsequently transferred for another 15 min to the ‘handling and restraint’ treatment (see below). Immediately after the experiment, individuals were transferred to a cage containing a heat patch for 10 min, to facilitate body temperature recovery after the temperature treatment.

In the handling and restraint treatment, the chick was subjected to a standardized ‘handling and restraint’ stress protocol (hereby ‘restraint’) widely used in previous studies (e.g. Bonier et al., 2004; Dietz et al., 2013; Jimeno et al., 2017; Taff et al., 2022). Chicks were taken out of the nest box and immediately introduced in an opaque and breathable cloth bag for 15 min to trigger an acute GC response. On the day of cumulative treatment, chicks spent 15 min in the restraint treatment, and were subsequently transferred to the temperature reduction treatment for the remaining 15 min.

Immediately after treatment completion of the treatments at days 3 and 4, 150 μl of blood was extracted from every nestling to determine glucose and corticosterone levels in response to the experimental manipulation. Blood glucose was measured immediately after collecting each blood as mentioned above. Blood samples were then kept cool and transferred to the lab on the same day (i.e. within 8 h), where they were centrifuged for 5 min at 9000 ***g*** to separate the plasma from red blood cells. Plasma was subsequently stored at −80°C until hormone extraction.

### Hormone analyses

A total of 190 blood samples were analyzed for corticosterone and 189 blood samples for glucose (two and three samples failed owing to insufficient plasma volume, respectively, and three more could not be taken owing to brood natural death before the second day of experiment). Corticosterone extraction was carried out following a previously described double extraction protocol with diethyl ether (Heidinger et al., 2010; Malisch et al., 2010). A total of 15 μl of plasma and 500 μl of diethylether were used for the extraction, and the extracted sample was resuspended in 200 μl of steroid-free serum (Arbor E6, cat. no. X065-28ML).

Plasma corticosterone levels were quantified by enzyme immunoassay (EIA) (cat. no. K017-H1/H5, Arbor Assays, DetectX^®^) following the manufacturer’s protocol. Extracted hormone samples (50 μl) were assayed in duplicate, and samples from the same individuals – and whenever possible from the same nest – were analyzed in the same plate. Two control samples – one with high corticosterone concentrations and another with low corticosterone concentrations – were added on each plate (*N*=6 plates) to obtain among-plate variability. Extraction efficiency as calculated by cold-spiking controls was high (91.64%) and we did not correct individually for sample-specific efficiency. The average intra-plate coefficient of variation was 9.38%, and the among-plate coefficient of variation was 12.92%.

### Statistical analysis

Statistical analyses were carried out using R 4.2.0 (https://www.r-project.org/), using the ‘lmer’ function of the ‘lme4’ package (Bates et al., 2015) and the ‘ggplot2’ package (Wickham, 2016) to develop the figures. All final models fitted assumptions of normality of residuals, normality of random effects, multicollinearity and homogeneity of variance (‘check_model’ function in the ‘performance’ package; Lüdecke et al., 2021). Glucose (mg dl^−1^) and corticosterone (ng ml^−1^) concentrations were normalized by logarithmic transformation (ln).

We first performed preliminary general linear mixed-effects models (GLMMs) to test for potential effects of environmental and methodological variables on glucose and corticosterone (at both baseline and experimentally induced levels) that may need to be added in the main models (see below). We then built specific models with three main objectives: (1) to assess the treatment effects on corticosterone and glucose levels (unique treatment); (2) to test whether the response patterns differed when the two stimuli occurred subsequently (unique treatment versus cumulative treatment); and (3) to test the correlations between glucose levels and corticosterone levels, within (i.e. responses) and among (i.e. before and after experimental manipulations) individuals. To further test the differences in glucose and corticosterone levels among experimental treatments, additional *post hoc* tests were performed (i.e. Tukey test using the ‘emmeans’ function; https://CRAN.R-project.org/package=emmeans).

#### Effect of methodological and environmental variables on glucose and corticosterone

##### Baseline levels (pre-treatment)

We carried out two GLMMs where either baseline glucose or baseline corticosterone was included as the dependent variable. Nest was included as a random factor, time (i.e. seconds) from first disturbance to the collection of the blood sample and time of day (hour of day) were included as covariates, and date was included as a categorical factor.

##### Response to experimental treatments (post-treatment)

We carried out two GLMMs where either glucose or corticosterone after treatment was included as the dependent variable. Nest and chick identity were included as random factors, initial temperature inside the nest box and handling sequence were introduced as covariates, and treatment order (i.e. whether cumulative treatment was applied on the day after unique treatment, or vice versa), first or second experimental day (i.e. day 3 or day 4) and whether the treatment was cumulative were included as categorical factors.

#### Effects of experimental treatment on glucose and corticosterone levels

##### Unique treatment

The effect of the experimental treatment was evaluated using two models in which glucose or corticosterone levels after the treatment were the dependent variables, nest identity was included as a random factor, initial nest temperature and body mass as covariates, and group treatment, experimental day (only in the glucose models) and sex were included as categorical factors.

##### Cumulative effects

We ran models as above for the whole dataset (i.e. including both unique and cumulative manipulations), this time including the variable ‘cumulative’ (i.e. ‘yes’ or ‘no’) as an additional variable, and both nest and chick identity as random effects. In these models, we additionally included the interaction between the treatment and the cumulative factor, in order to test whether glucose or corticosterone response patterns differed when treatments occurred subsequently.

#### Correlations between corticosterone and glucose

Correlations between corticosterone and glucose levels from the same blood samples were evaluated at three levels, using general linear models.

##### Among-individual, baseline

Baseline glucose was included as the dependent variable, nest identity as a random effect and baseline corticosterone as a predictor.

##### Among-individual, experimentally induced

Glucose levels after both cumulative and unique experimental treatments (i.e. complete dataset) was included as the dependent variable; corticosterone levels were included as the predictor variable; nest and chick identity were included as random effects; group treatment, sex, experimental day and cumulative were included as categorical factors; and initial nest temperature and chick body mass were included as covariates.

##### Within-individual

We built up a model in which the difference between the glucose levels in response to experimental treatments (both cumulative and unique) and baseline glucose levels taken on day 2 were included as the dependent variable, and as independent variable we included the difference between the corticosterone levels in response to experimental treatments and the baseline corticosterone levels. Nest and chick identities were included as random factors.

## RESULTS

### Effect of methodological and environmental variables on glucose and corticosterone

**Fig. 2.**
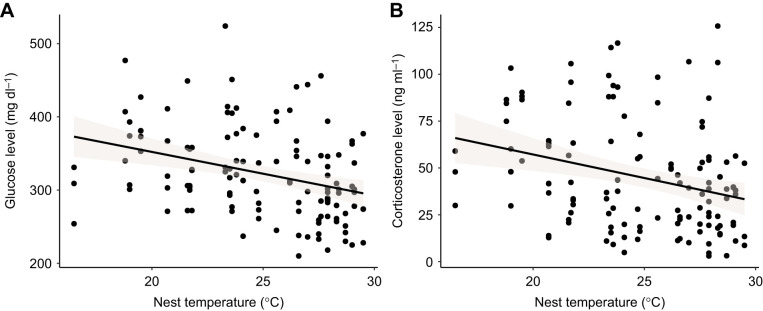
**Relationship between the temperature (°C) inside the nest box prior to manipulation and glucose or corticosterone levels (ln transformed) after the treatments.** (A) Glucose; (B) corticosterone. Each panel includes two measurements per individual corresponding to the two experimental days (*N*=65 individuals), but note that the statistical models correct for individual identity as a random factor.

None of the environmental and methodological variables considered (seconds from disturbance until collection of blood samples, date and hour) had a significant effect on baseline glucose and corticosterone levels (Table S1a). In contrast, initial nest temperature measured prior to manipulation had a significant, negative effect on both glucose and corticosterone levels obtained after the experimental manipulations (Fig. 2; Table S1b, Fig. S2). In addition, we found a significant and positive effect of the factor ‘day’ (i.e. if it was the first or second day of experiment), with higher glucose levels on the second day of the experiment (Table S1b).

#### Effects of experimental treatment on glucose and corticosterone levels

##### Unique treatment

We found a significant, positive effect of experimental treatments on both glucose and corticosterone levels, whose magnitude differed among treatment groups (Table S2). Individuals in the temperature reduction treatment showed higher glucose and corticosterone levels as compared with handling control individuals (glucose: *t*_41.20_=−4.98, *P*≤0.001; corticosterone: *t*_43.80_=−4.71, *P*≤0.001). They also showed a non-significant tendency to have higher glucose levels as compared with individuals in both the restraint and disturbance control treatments (restraint: *t*_40.20_=−2.51, *P*=0.07; disturbance control: *t*_40.20_=−2.55, *P*=0.07). Individuals in the restraint and disturbance control treatments showed higher corticosterone levels as compared with individuals in the handling control treatment (*t*_41.80_=−5.08, *P*=<0.001 and *t*_43.50_=−2.74, *P*=0.04, respectively).

##### Cumulative effects

**Fig. 3.**
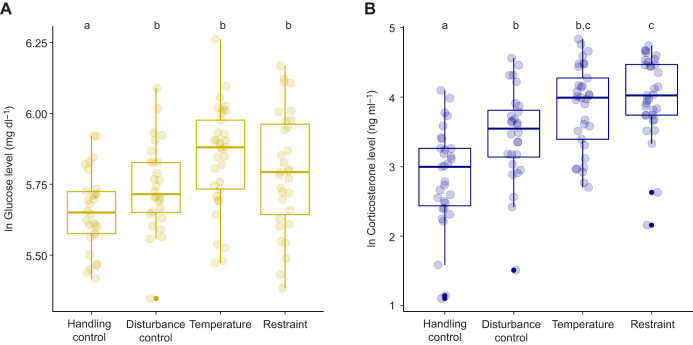
**Differences in glucose and corticosterone levels (ln transformed) as a function of experimental treatment including the complete dataset (cumulative+unique).** (A) Glucose; (B) corticosterone. The bottom and top lines of the box represent the interquartile range, and the horizontal line inside the box represents the median. The whiskers represent values outside the lower and upper quartile. Different letters above the boxplots indicate significant differences according to Tukey's *post hoc* tests. Handling control: the chick stayed undisturbed; disturbance control: the chick was transferred to a nest box similar to the one used in the temperature reduction treatment to mimic experimental conditions; Temperature: temperature reduction treatment (the chick was placed in a nest box equipped with a temperature manipulation device); Restraint: the standardized stress response treatment (the chick was introduced in an opaque and breathable cloth bag).

Whether the experimental treatment was unique or cumulative did not have an effect on glucose or corticosterone responses (glucose model: *F*_1,96.96_=0.67, *P*=0.41; corticosterone model: *F*_1,52.72_=2.76, *P*=0.1; Table 1, Fig. 3; Fig. S3), nor did the interaction between the factor ‘cumulative’ and the treatment group [treatment×cumulative (glucose model) *F*_3,89.61_=1.00, *P*=0.39; treatment×cumulative (corticosterone model) *F*_3,50.65_=1.09, *P*=0.36; Table 1]. Including the cumulative data did not cause qualitative differences on the treatment effects on glucose and corticosterone (Table 1, Fig. 3), only leading to changes (i.e. overall increases) in the magnitude of such effects (Fig. 3). In contrast to the previous model not including the cumulative experiments, individuals in both the restraint and disturbance control treatments showed higher glucose values as compared with individuals in the handling control treatment (restraint: *t*_42.00_=−3.96, *P*<0.01; disturbance control: *t*_40.00_=−2.78, *P*=0.04). Similarly, individuals in the restraint treatment showed higher corticosterone values as compared with individuals in the disturbance control treatment (*t*_41.90_=−2.86, *P*=0.03).

**Table 1.**
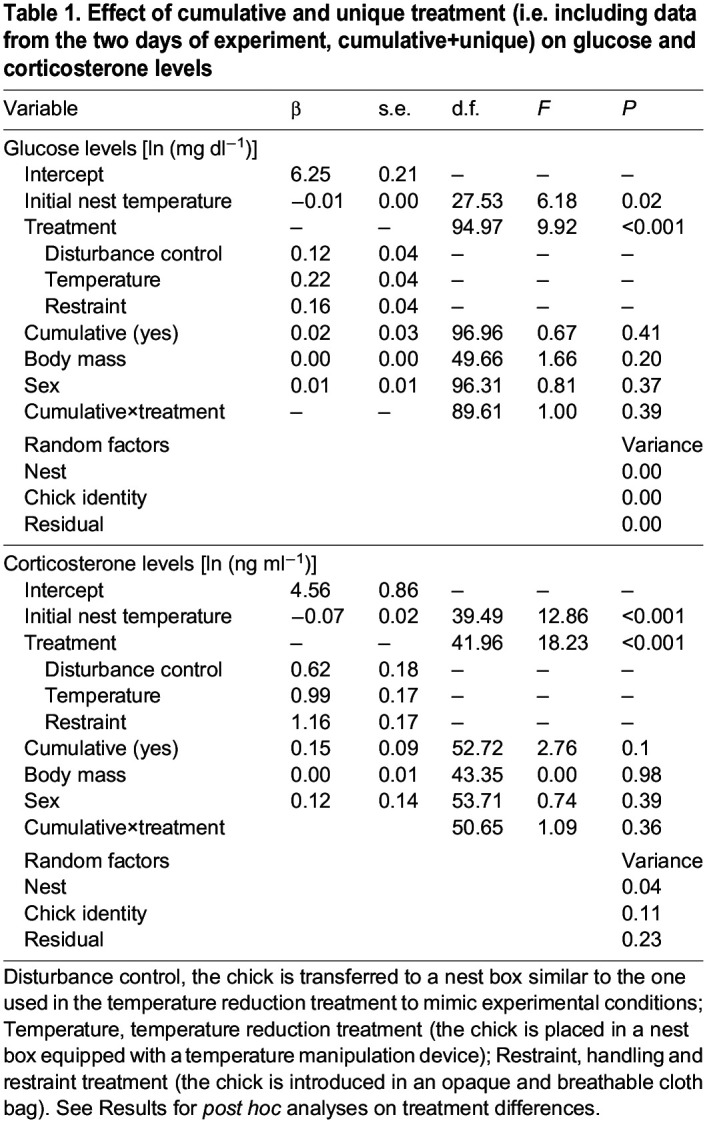
Effect of cumulative and unique treatment (i.e. including data from the two days of experiment, cumulative+unique) on glucose and corticosterone levels

#### Correlation between corticosterone and glucose

**Fig. 4.**
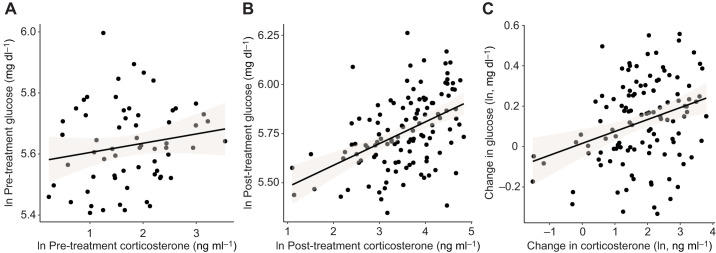
**Correlations between glucose and corticosterone levels (ln transformed).** (A) Relationship between pre-treatment glucose levels and pre-treatment corticosterone levels. (B) Relationship between post-treatment glucose levels and post-treatment corticosterone levels. (C) Relationship between the change in glucose levels (ln post- minus ln pre-treatment) and the change in corticosterone levels (ln post- minus ln pre-treatment). B and C include more than one data point for each individual (unique+cumulative treatments), but note that the statistical models correct for individual identity that is considered as a random term.

We did not find a correlation among individuals between baseline glucose and corticosterone levels (β=0.03, *F*_1,58.98_=2.25, *P*=0.14; Fig. 4A). In contrast, glucose and corticosterone levels after experimental treatments were significantly correlated among individuals (β=0.05, *F*_1,107.52_=4.54, *P*=0.04; Fig. 4B). We also found a significant correlation within individuals between the change (post- minus pre-treatment) in glucose levels and the change (post- minus pre-treatment) in corticosterone levels (β=0.09, *F*_1,95.36_=15.94, *P*<0.001; Fig. 4C).

## DISCUSSION

Blood GCs have been shown to increase in response to environmental disturbances to help vertebrates restore homeostasis and overcome the challenge. This observation has led to consider GCs as ‘stress hormones’, often overlooking their sustained metabolic function (Koolhaas et al., 2011; McDougall-Shackleton et al., 2019; Jimeno and Verhulst, 2023) and the role that environmental factors affecting energy expenditure may have on GC measurements. It has been hypothesized that natural GC variation responds to changes in energetic needs and that acute GC responses facilitate the energetic boost needed to overcome (or recover from) an increase in metabolic rate (Jimeno and Verhulst, 2023). Therefore, we tested the effect of two stimuli of different nature, known to increase metabolic rate and GCs, respectively (i.e. temperature and restraint), on plasma corticosterone (the main avian GC) and glucose (the main bird fuel substrate). In the experiment we also included two control treatments, to account for the effects that (1) manipulating the nest box and (2) handling individuals may have on the two study variables. Our results show that both experimentally induced temperature decrease and restraint treatments caused a similar increase in corticosterone and glucose. Furthermore, the responses to the four treatment groups (disturbance control, handling control, temperature reduction and restraint) followed comparable patterns for corticosterone and glucose, so that the two experimental groups showed the highest values, followed by the disturbance control and the handling control.

Despite the similarity of these patterns, there were some differences between the two study variables in the response to the experimental manipulations. We found that corticosterone levels after restraint were similar to those after the temperature reduction treatment (and significantly higher than those after both controls). By contrast, glucose levels after restraint showed a non-significant tendency to be lower than those after the temperature reduction treatment (*P*=0.08), and remained similar to those after the disturbance control treatment (Fig. 3). Individuals in the disturbance control treatment consistently showed significantly higher corticosterone and glucose levels than those in the handling control. Glucose regulation is complex and accomplished through multiple routes. In the context of acute responses, the main short-term increase in circulating glucose results from a decrease in glucose utilization (Romero and Beattie, 2021), along with an immediate response mediated by catecholamines (i.e. adrendaline and noradrenaline) and glucagon, which initiate glycogenolysis (Jones et al., 2012). Catecholamines also inhibit insulin release (Taborsky and Porte, 1981) and mobilize fat breakdown (Wajchenberg et al., 2002), further helping to supply energy. A rise in glucagon may also interact with the fall in insulin to facilitate fuel availability to meet increased energy demand (reviewed in Jones et al., 2012). These short-term processes, rather than GC actions, may underlie the results that we found for glucose, so that the time interval we considered (i.e. 15 min) would not be long enough to capture GC-induced increases in glucose, or only partially. This could explain why we did not find statistical differences in glucose levels between the experimental groups involving chick handling (i.e. all but handling control), and thus presumably adrenaline release (Fig. 3; Deviche et al., 2016b). In contrast, in light of the observed patterns (Fig. 3), it seems clear that corticosterone and glucose concentrations in the disturbance control consistently tend to remain lower than those in the experimental treatments, likely because of the higher and sustained energetic demands posed by the two experimental stimuli. Thus, the lack of statistical significance in some of these comparisons could also be explained by a modest sample size, but further experiments would be required to confirm or reject this interpretation.

Our results suggest that both corticosterone and glucose increase when individuals face environmental challenges entailing an increase in energy demand, regardless of the nature of the disturbance, supporting the key role of energy metabolism on GC variation, also during acute GC responses. Although we cannot prove that the response patterns we found would hold when applying other stimuli differing in nature or intensity, the generality of the above relationship is supported by previous studies finding an association between GCs and metabolic rate under controlled conditions (Beerling et al., 2011; Buwalda et al., 2012; Jimeno et al., 2018; Malkoc et al., 2021), or between GCs and variables directly related to metabolic rate under natural conditions, such as ambient temperature (Jessop et al., 2016). Furthermore, several comparative studies investigating the association between metabolism and GCs across bird and mammal species also found such an association (Haase et al., 2016; Rubalcaba and Jimeno, 2022; Jimeno and Verhulst, 2023; but see Francis et al., 2018), but note that none of the above studies measured glucose levels. Interestingly, in this study, we found a strong and negative association between both glucose and corticosterone and ambient temperature inside the nest box prior to experimental manipulations (Fig. 2), which reinforces the metabolic dependence of GCs, as lower temperatures require a higher energy expenditure and glucose demand to maintain body temperature (Montoya et al., 2018).

Given that GC levels are often assumed to provide information on organismal stress and physiological status, one could argue that all our experimental manipulations (including the disturbance control) triggered a psychological stress response that differed in magnitude among treatment groups. This would question the direct association between GCs and metabolic demands, as it is often assumed that psychological stress may trigger a peak in GC secretion, with or without an immediate increase in energy expenditure. Although we cannot rule out this hypothesis – as we did not directly measure metabolic rate – we consider the causal link between metabolic rate and GCs the most parsimonious explanation because (1) corticosterone and glucose changes and levels after experimental manipulations were strongly correlated and (2) the magnitude of the increase in both variables was higher as the metabolic challenge increased. These results support recent evidence on the direct associations between GCs and energy expenditure (Jimeno and Verhulst, 2023; Malkoc et al., 2021; Jimeno et al., 2018), and thus we suggest that a causal link between metabolic rate and GCs is the most parsimonious explanation to our findings.

We hypothesized that mounting a metabolic and GC response may prevent individuals from mounting or adjusting an additional one to a subsequent stimulus. To test this, we analyzed whether glucose and corticosterone responses differed when both experimental treatments occurred subsequently in time. In line with this hypothesis, we found that exposing individuals to an additional stimulus of different nature (i.e. additional 15 min) did not cause a change in corticosterone and/or glucose levels or response patterns. This was also true for the two control treatments, in which individuals remained longer (i.e. 30 min instead of 15 min). Although we found some quantitative statistical differences among glucose and corticosterone values in response to experimental manipulations when including the data from the cumulative treatment day, the overall patterns and differences among treatments remained the same. We therefore attribute these small differences to the increase in statistical power when including the cumulative data, as sample size was doubled when considering a second sample of all individuals. An alternative explanation to the existence of this limitation in the response is that the time scale considered was insufficient to detect an additional response. In this study, we tried to minimize the disturbance time by experimental manipulations on chicks (i.e. 15 min treatments and 30 min maximum; Taff et al., 2022), but it is possible that considering a longer time scale would have allowed us to detect an additional response to the second stimulus in the cumulative treatment data. Indeed, a majority of the studies investigating acute GC responses take GC measurements at 30 or even 45 min after first disturbance (e.g. Love et al., 2003; Wada et al., 2009; Jimeno et al., 2017). However, the time interval to reach the peak in GC secretion highly varies across species, being often 20–30 min in birds (Romero and Butler, 2007), and sometimes as soon as 10 min (Deviche et al., 2016a). Because stress-induced responses may vary in magnitude as a function of the intensity of the trigger and/or how the stimulus is perceived by the organism (Schoenle et al., 2018), it would be interesting to test whether these patterns remain when triggering a maximum physiological response, for instance by including an experimental treatment in which ACTH (adrenocorticotropic hormone) is administrated (Jimeno et al., 2017; MacDougall-Shackleton et al., 2019).

Whereas there is an often implicit assumption that a stronger GC response to a stimulus will result in similarly strong downstream effects (including a larger increase in glucose; Romero and Gormally, 2019), this has rarely been tested. We found a strong correlation between glucose and corticosterone, also at the within-individual level, which holds independently of experimental treatment and whether the individual was exposed to a single or two subsequent stimuli. In line with our results, a significant association between changes in plasma glucose and corticosterone as soon as 10 min after standardized stress (but not after 30 min) was also found in rufous-winged sparrows (Deviche et al., 2016a). By contrast, we did not find a correlation between glucose and corticosterone at baseline levels. This latter result is supported by a recent study measuring glucose and corticosterone in free-living tree swallow nestlings and adults (Taff et al., 2022). The lack of correlation between the baseline glucocorticoid and glucose levels may be due to individuals differing in the basal rate at which the organism operates. This among-individual variability would be reduced when exposing individuals to a specific stimulus increasing energetic demands, so that individuals responding with a higher increase in plasma GCs would consequently exhibit a higher increase in plasma glucose. However, the study by Taff et al. (2022) did not find an association between the increases in glucose and corticosterone in response to a standardized stressor, as we did. There are several explanations that could underlie this difference. It could be that GCs and glucose are tightly coupled within individuals, but that within-individual correlations are not detected at a between-individual level (Agrawal et al., 2020; Taff et al., 2022). Furthermore, as GC secretion and dynamics are tightly regulated by HPA components such as receptors (Lattin et al., 2015; Jimeno and Zimmer, 2022), individuals may differ in GC sensitivity so that producing a similar physiological response (i.e. change in glucose) may require different levels of GCs in different individuals (Jimeno and Zimmer, 2022; Taff et al., 2022; Jimeno and Rubalcaba, 2024). The interpretation of glucose availability during or after an acute GC response remains debated (Romero and Beattie, 2021; Taff et al., 2022), mostly due to glucose being regulated by coexisting shorter- (i.e. catecholamines and glucagon) and longer-term (i.e. GCs) hormones. Whereas the strong correlation between GCs and glucose during an acute response may suggest a causal relationship between the two variables, we cannot rule out that the increases in glucose we detected were caused by other (short-term) regulatory components. If the former, GC-induced increases in glucose may be interpreted as an allocation adjustment to the metabolic level at which organisms operate, in line with the role of GCs mediating glucose synthesis and mobilization to help the organism reallocate resources and restore homeostasis (Jimeno and Verhulst, 2023). If the latter, the time interval considered in our experiments (15 to 30 min) would not have been long enough to detect GC-induced hyperglycemia, as GCs are produced *de novo* in the adrenal gland and need to bind to receptors to exert their actions (Herman, 2022; Romero and Beattie, 2021). Further research investigating GC and glucose dynamics within acute responses and accounting for other hormones (i.e. catecholamines, insulin and glucagon), are needed to understand the relative contribution of GCs to short- versus longer-term hyperglycemia.

Our results add to the increasing research on the role of energy expenditure in GC variation. Our experimental evidence provides support to the association between plasma GCs and energy metabolism (i.e. glucose levels) in a free-living species, also during acute responses and across environmental challenges. Although our experimental design does not allow us to infer causal associations between corticosterone and glucose, the strong correlations found between these traits in response to our experimental treatments show that both glucose and corticosterone levels reflect the magnitude of increases in energy expenditure.

## Supplementary Material

10.1242/jexbio.246905_sup1 Supplementary information
