# Supplementary material for: Corticosterone and glucose are correlated and show similar response patterns to temperature and stress in a free-living bird
Source: J Exp Biol. 2024 Jul 24;227(14):jeb246905. doi: 10.1242/jeb.246905 (PMC11418182; doi:10.1242/jeb.246905)
Supplement: Supplementary information [file jexbio-227-246905-s1.pdf]

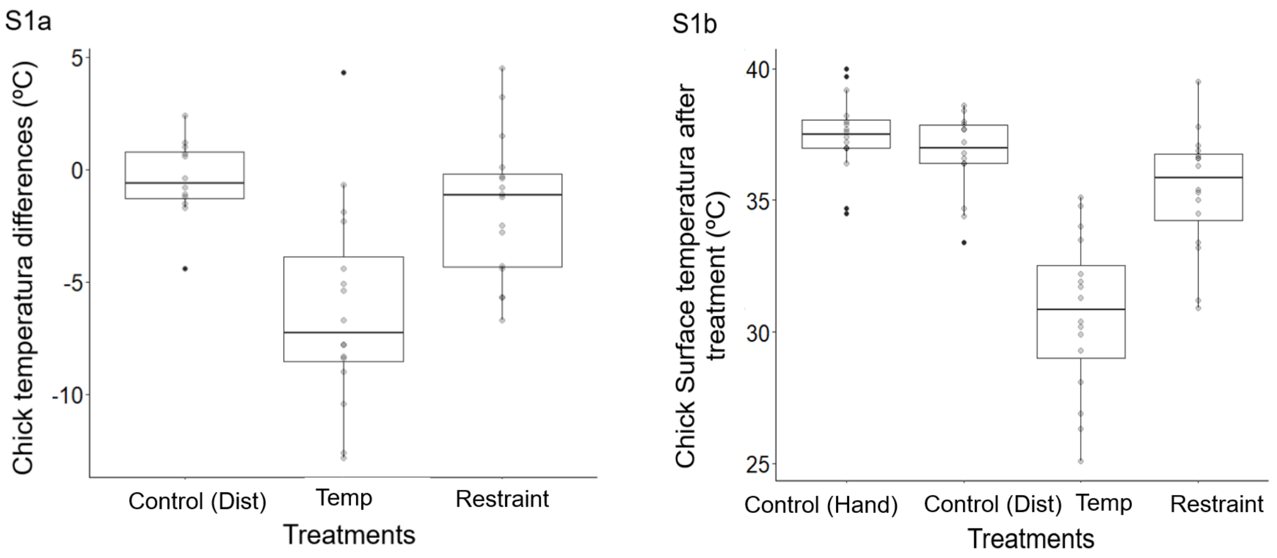

Fig. S1. Chick temperature change (ºC; chick surface temperature after treatment – chick surface temperature before treatment) as a function of treatment (a) and difference in mean chick surface temperature after treatment as a function of treatment type (b). Where Control (Hand) is the handling control (The chick stayed undisturbed); Control (Dist) is the disturbance control (the chick was transferred to a nest box similar to the one used un the temperature reduction treatment to mimic experimental conditions); Temp is the temperature reduction treatment (the chick was placed in a nest box equipped with a temperature manipulation device) and Restraint is the handling and restraint treatment (the chick was introduced in an opaque and breathable cloth bag).

Journal of Experimental Biology • Supplementary information

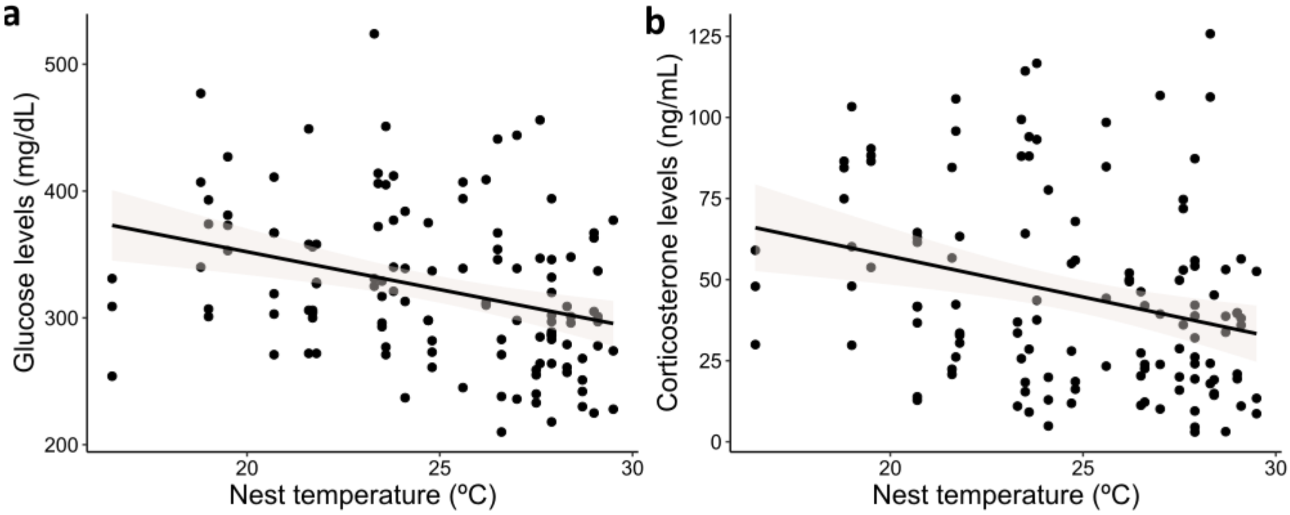

Fig. S2. Relationship between the temperature (ºC) inside the nest box prior to manipulation and glucose (a) or corticosterone (b) levels (untransformed) after the treatments. Each panel includes two measurements per individual corresponding to the two experimental days (N = 65 individuals) but note that the statistical models correct for individual identity as a random factor.

Journal of Experimental Biology • Supplementary information

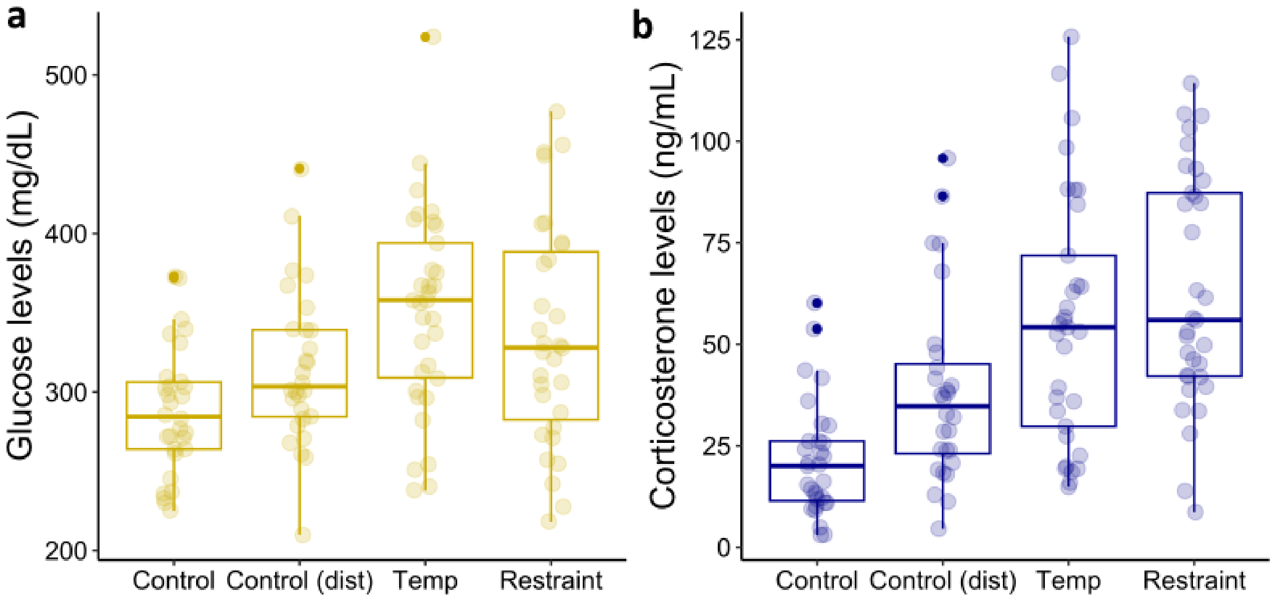

Fig. S3. Differences in glucose (a) and corticosterone (b) levels (untransformed) as a function of experimental treatment including the complete dataset (cumulative + unique). The bottom and top lines of the box represent the interquartile range, and the horizontal line inside the box represents the median. The whiskers represent values outside the lower and upper quartile. Different letters above the boxplots indicate significant differences according to Tukey's post hoc tests. Control (hand) is the handling control (the chick stayed undisturbed), Control (Dist) is the disturbance control (the chick was transferred to a nest box similar to the one used un the temperature reduction treatment to mimic experimental conditions), Temp is the temperature reduction treatment (the chick was placed in a nest box equipped with a temperature manipulation device) and Restraint is the standardized stress response treatment (the chick was introduced in an opaque and breathable cloth bag).

Journal of Experimental Biology • Supplementary information

Table S1. Effect of environmental and methodological variables on pre-treatment (a) and post-treatment (b) glucose and corticosterone levels.

a)

Pre-treatment glucose levels (Ln (mg/dL))					
**Variables**	**β**	**SE**	**d.f.**	**F**	**P**
Intercept	2.47	0.09	-	-	-
Seconds until blood sample	0.00	0.00	54.77	0.04	0.85
Date	-	-	11.98	2.73	0.07
Hour	0.00	0.00	13.32	0.23	0.64
**Random factors**					**Variance**
Nest					0.00
Residual					0.00

Pre-treatment corticosterone levels (Ln (ng/mL))					
**Variables**	**β**	**SE**	**d.f.**	**F**	**P**
Intercept	0.71	0.65	-	-	-
Seconds until blood sample	0.00	0.00	54.15	1.89	0.17
Date	-	-	9.99	0.63	0.68
Hour	0.01	0.03	10.72	0.14	0.72
**Random factors**					**Variance**
Nest					0.05
Residual					0.07

b)

Post-treatment glucose levels (Ln (mg/dL))

**Variables**	**β**	**SE**	**d.f.**	**F**	**P**
Intercept	6.19	0.16	-	-	-
Treatment day	0.06	0.03	61.38	4.29	0.04
Nest temperature	-0.02	0.01	29.87	11.07	<0.01
Treatment order	-0.01	0.05	15.62	0.06	0.81
Cumulative (YES)	0.02	0.03	57.16	0.43	0.51
Sequence	0.01	0.01	105.2	0.25	0.62
**Random factors**					**Variance**
Chick identity					0.00
Nest					0.00
Residual					0.02

Post-treatment corticosterone levels (Ln (ng/mL))

**Variables**	**β**	**SE**	**d.f.**	**F**	**P**
Intercept	5.51	0.58	-	-	-
Treatment day	-0.09	0.09	65.72	0.99	0.32
Nest temperature	-0.08	0.02	118.71	13.91	<0.001
Treatment order	-0.26	0.19	67.49	1.93	0.17
Cumulative (YES)	0.17	0.09	59.18	3.55	0.06
Sequence	0.05	0.05	93.5	0.86	0.36
**Random factors**					**Variance**
Nest					0.00
Chick identity					0.33
Residual					0.22

Journal of Experimental Biology • Supplementary information

Table S2. Effect of experimental treatments (unique treatment only) on glucose (Ln (mg/dL)) and corticosterone (Ln (ng/ml)) levels. Where Control (Dist) is the disturbance control (the chick is transferred to a nest box similar to the one used un the temperature reduction treatment to mimic experimental conditions); Temp is the temperature reduction treatment (the chick is placed in a nest box equipped with a temperature manipulation device) and Restraint is the handling and restraint treatment (the chick is introduced in an opaque and breathable cloth bag).

Glucose levels (Ln (mg/dL))

Variables	β	SE	d.f.	F	P
Intercept	6.7	0.27	-	-	-
Nest temperature	-0.03	0.01	14.1	12.21	<0.001
Treatment day	0.05	0.05	13.2	1.03	0.33
Treatment	-	-	40.44	8.33	<0.001
Control (Dist)	0.12	0.05	-	-	-
Temp	0.23	0.05	-	-	-
Restraint	0.11	0.05	-	-	-
Weight	-0.01	0	31.2	3.55	0.07
Sex	-0.003	0.04	51.27	0.01	0.93
**Random factors**					**Variance**
Nest					0.005
Residual					0.02

Corticosterone levels (Ln (ng/mL))

Variables	β	SE	d.f.	F	P
Intercept	4.48	1.19	-	-	-
Box temperature	-0.08	0.03	16.07	6.72	0.02
Treatment	-	-	44.16	10.7	<0.0001
Control (Dist)	0.72	0.26	-	-	-
Temp	1.2	0.25	-	-	-
Restraint	1.25	0.24	-	-	-
Weight	0.00	0.02	31.32	0.03	0.86
Sex	0.03	0.19	54.97	0.02	0.89
**Random factors**					**Variance**
Nest					0.04
Residual					0.47
